# The Yin and Yang of Invariant Natural Killer T Cells in Tumor Immunity—Suppression of Tumor Immunity in the Intestine

**DOI:** 10.3389/fimmu.2017.01945

**Published:** 2018-01-10

**Authors:** Ying Wang, Susanna L. Cardell

**Affiliations:** ^1^Department of Microbiology and Immunology, University of Gothenburg, Gothenburg, Sweden

**Keywords:** natural killer T cells, CD1d, tumor immunity, immunosuppression, intestinal inflammation, intestinal polyposis

## Abstract

CD1d-restricted invariant natural killer T (iNKT) cells are known as early responding, potent regulatory cells of immune responses. Besides their established role in the regulation of inflammation and autoimmune disease, numerous studies have shown that iNKT cells have important functions in tumor immunosurveillance and control of tumor metastasis. Tumor-infiltrating T helper 1 (TH1)/cytotoxic T lymphocytes have been associated with a positive prognosis. However, inflammation has a dual role in cancer and chronic inflammation is believed to be a driving force in many cancers as exemplified in patients with inflammatory bowel disease that have an increased risk of colorectal cancer. Indeed, NKT cells promote intestinal inflammation in human ulcerative colitis, and the associated animal model, indicating that NKT cells may favor tumor development in intestinal tissue. In contrast to other cancers, recent data from animal models suggest that iNKT cells promote tumor formation in the intestine by supporting an immunoregulatory tumor microenvironment and suppressing TH1 antitumor immunity. Here, we review the role of iNKT cells in suppression of tumor immunity in light of iNKT-cell regulation of intestinal inflammation. We also discuss suppression of immunity in other situations as well as factors that may influence whether iNKT cells have a protective or an immunosuppressive and tumor-promoting role in tumor immunity.

## Introduction

CD1d-restricted natural killer T (NKT) lymphocytes belong to a diverse group of non-conventional T cells that recognize non-peptide antigens. These T cells have some general features that distinguish them from conventional T cells that are activated by foreign peptides presented on MHC class-I and -II molecules (1, 2). Non-conventional T cells often have a memory/pre-activated phenotype and respond more rapidly to stimulation and can be activated in the absence of T-cell receptor (TCR) signals. They are generally not recirculating but preferentially localize to particular tissues and have a reduced TCR diversity compared with conventional T cells. These characteristics make non-conventional T cells rapid responders to infection, inflammatory signals, and tissue damage and enable them to regulate the quality and quantity of immune responses. Recent progress in our understanding of the activation and function of these cells has led to an increased appreciation of their role in health and disease. The evolutionary conservation of CD1d and NKT cells between mouse and humans, and the essentially non-polymorphic nature of CD1d, has made the CD1d-NKT-cell system attractive to explore for the development of targeted immunomodulation.

Natural killer T cells recognize lipid and glycolipid antigens of self or microbial origin presented on the MHC class-I-like CD1d molecule. The term “NKT cells” is now generally used synonymously with CD1d-restricted T cells that carry TCRαβ (3). This definition will be used here. NKT cells can be divided into two subsets based on the TCR expressed. The first is type I or invariant NKT (iNKT) cells. These display an invariant TCR α-chain (Vα14-Jα18 in the mouse, Vα24-Jα18 in humans) paired with TCR β-chains using a limited set of Vβ segments but diverse CDR3 (4–6). In contrast, type II or diverse NKT (dNKT) cells have diverse TCR (7–9). The relative roles of these NKT-cell subsets have been explored in mice lacking all NKT cells (*CD1d*^−/−^ mice) and mice lacking only iNKT cells (*Jα18*^−/−^ mice). Studies of iNKT cells have also been greatly facilitated by the use of CD1d multimers loaded with the iNKT-cell ligand α-galactosylceramide (αGalCer) (10) which detects essentially all iNKT cells with high specificity. In contrast, while the role of dNKT cells can be studied in TCR transgenic mice (11) and deduced from comparisons of *CD1d*^−/−^ and *Jα18*^−/−^ mice, there are no specific reagents that detect all dNKT cells. Thus, studies of dNKT cells are more challenging. From studies of animal models and humans, both subsets of NKT cells have been suggested to play a role in diverse immune settings including autoimmunity, immunity to infections, and tumor immunity. Sometimes the two subsets have a similar function, while in other immune responses they counteract each other (12, 13). Besides the division of NKT cells based on their TCR, different functional programs have been identified in iNKT cells (14). For example, the iNKT1, iNKT2, and iNKT17 subsets express distinct sets of transcription factors. This makes them poised for the production of certain cytokines analogous to the T helper (TH) 1, TH2, and TH17 conventional T-cell subsets and corresponding subsets of innate lymphoid cells. More recently, iNKT cells with immunosuppressive function have been described that do not fit into this classification, as discussed below (15–17).

**Figure 1:**
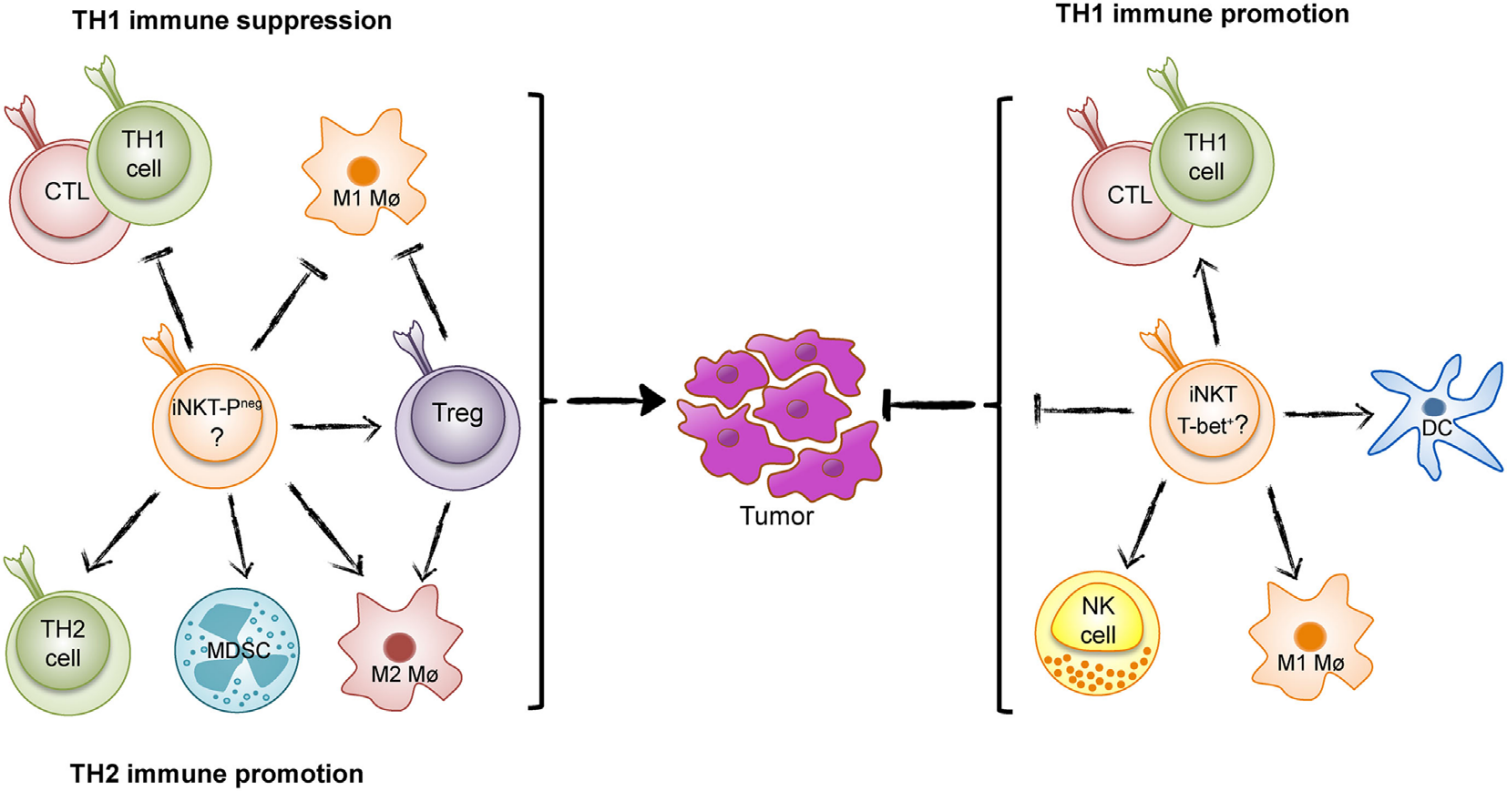
The Yin and Yang of invariant natural killer T (iNKT) cells in tumor immunity. iNKT cells can either suppress T helper 1 (TH1) tumor immunity and promote an immunosurveillance environment as found in intestinal polyposis (Yin, left) (PLZF-neg; PLZF-negative), or enhance TH1 immunity to tumors as found in many tumor models and inferred from studies of human cancer (Yang, right).

The majority or data from animal models and inferred from studies of human cancers show that iNKT cells enhance TH1 tumor immunity and combat tumors. However, in some situations, iNKT cells have surprisingly have demonstrated the opposite effect and promoted tumor development (Figure 1) (17–19). A recent example of iNKT-cell promotion of tumors is from a spontaneous mouse model for human colon cancer (17). We recently demonstrated that deletion of iNKT cells in this model reduces spontaneous intestinal polyp formation by 75%. Here, we will discuss our findings in light of the role of iNKT cells in intestinal immunity and compare tumor models that demonstrate tumor suppressive and tumor-promoting effects of iNKT cells.

## NKT Cells Enhancing Tumor Immunity in Many Models

The potent role of iNKT cells in tumor immunosurveillance and suppression of tumor metastasis was demonstrated many years ago. The enhancement of tumor immunity by iNKT cells has been described in several publications and discussed in many reviews [see for example Ref. (20)], and has led to intense studies aimed at targeting iNKT cells to combat cancer. The strong agonist iNKT-cell ligand αGalCer very efficiently activates iNKT cells leading to reduction or eradication of tumors in several animal models (13). Subsequent studies have addressed iNKT-cell function in tumor immunity after treatment with αGalCer and structural variants (21). iNKT cells can directly kill cancer cells, but the most important role of iNKT cells may be to activate other immune cells involved in tumor immunity. This includes activation of NK cells, maturation, and activation of dendritic cells, promotion of cytotoxic T cells and TH1 cells, and generally relies on the production of IFN-γ and TH1-related cytokines (Figure 1). iNKT cells can also counteract immunosuppressive cells. In patients, there is a reduced number of circulating iNKT cells in some cancers, and this reduction has been associated with poor survival in head and neck cancer and acute myeloid leukemia. These findings have led to clinical trials with iNKT-cell-directed immunotherapy in human cancer (22). In addition, iNKT cells perform natural tumor immunosurveillance in some animal models, suppressing the appearance of tumors and leukemia even in the absence of treatments that activate iNKT cells (23–26). In contrast to the usual protective function of iNKT cells, a natural immunosuppressive effect has been described for dNKT cells that promote tumor growth (27, 28).

## iNKT Cells Suppressing Tumor Immunity in Murine Intestinal Polyposis

The influence of NKT cells on tumor immunity or tumor growth and metastasis has frequently been investigated in tumor cell transplantation models such as the B16 melanoma model. These models have some advantages, including a coordinated initiation of growth, the availability of well-characterized tumor cell lines for transplantation as well as the possibility to genetically alter the tumor cells and design specific tools to study the tumor immune response. On the other hand, induction of tumors by injection of cell lines has the disadvantages of lacking the early steps of natural cancer initiation and the natural heterogeneity of tumors. Tumor growth is also usually at a site different from the origin of the tumor. These parameters may influence the immune response to the tumor, and the regulation of tumor immunity.

It is well known that cancer is promoted by inflammation. For example, the chronic inflammation in patients with inflammatory bowel disease (IBD) leads to increased risk for colorectal cancer. It has been described that iNKT cells promote inflammation in a mouse model for IBD (29), suggesting that iNKT cells might promote tumor formation in the intestine. To investigate this, we have studied iNKT-cell-mediated regulation of tumor immunity in the orthotopic spontaneous model for early stages of intestinal cancer, the *Apc*^min/+^ mouse (30). *Apc*^min/+^ mice are heterozygous for a truncating mutation in the adenomatous polyposis coli (*Apc*) tumor suppressor gene, and spontaneously develop polyps in the small and large intestines (31). The APC protein is part of an inhibitory complex associated with the canonical Wnt signaling pathway that ubiquitously controls cell differentiation and proliferation. Mutation of the *APC* gene is an early event in 80% of sporadic colorectal cancers in humans and is the mutated gene inherited in familial adenomatous polyposis. In the *Apc*^min/+^ mouse, polyps develop following additional genetic events such as loss of heterozygosity of the *Apc* gene (31).

Using mice deficient in either all NKT cells or specifically lacking iNKT cells, we found a dramatic reduction of intestinal polyps, demonstrating that iNKT cells favor polyp growth in this tissue (17). Detailed investigation of immune parameters revealed that iNKT cells suppressed the expression of genes associated with TH1 immunity, including IFN-γ, inducible nitric oxide synthase (iNOS), IL-12p40, T-bet, and granzyme B. A TH1-type immune response has been shown to prevent tumors in the *Apc*^min/+^ mouse model as well as in human colorectal cancer (32–34). In contrast, the presence of iNKT cells increased the proportion and activation of FoxP3-expressing regulatory T (Treg) cells specifically in polyps (17). The infiltration of high proportions of Treg cells in tumors is generally associated with suppressed tumor immunity and poor prognosis in cancer patients (35). Although Treg cells have a complex role in colon carcinogenesis, likely reflecting different functions of Treg cells at different stages of disease (36), deletion of Treg cells in the *Apc*^min/+^ mouse resulted in enhanced accumulation of conventional CXCR3^+^ T lymphocytes in polyps (37). Moreover, iNKT cells augmented the proportion of M2-like macrophages, a cell type that is associated with tumor promotion. They also elevated the numbers of myeloid-derived suppressor cells, especially those with a polymorphonuclear phenotype (17). Taken together, we concluded that iNKT cells support an immunosuppressive microenvironment, most pronounced in the polyps, and directly and/or through the promotion of Treg cells, inhibited a TH1 antitumor immune response (Figure 1).

It should be noted that the immune microenvironment changes in tumors of colon cancer as the disease progresses. The role of Treg cells in human colon cancer has been controversial, and different studies have associated high densities of infiltrating Treg cells with either a better or worse outcome. A possible explanation for this was recently provided, as it was demonstrated that at later stages of colon cancer, Treg cells become positive for the transcription factor RORγt and acquire the capacity to produce the tumor-promoting cytokine IL-17 (36). RORγt-expressing Treg cells retained the capacity to suppress T lymphocytes, but were no longer anti-inflammatory. Similar Treg cells were found in the *Apc*^min/+^ mouse, and ablation of RORγt in Treg cells in this model resulted in improved suppression of inflammation and attenuated polyposis. A study of human colorectal cancer demonstrated an increased infiltration of cells positive for Vα24, the TCR α-segment used by human iNKT cells, in carcinomas compared with unaffected tissue (38). A higher density of Vα24^+^ cells correlated with increased patient survival, suggesting that iNKT cells may be protective. This is in contrast to the findings in *Apc*^min/+^ mice where iNKT cells promote polyps. Further studies, using specific reagents such as αGalCer-loaded CD1d multimers, are required to investigate whether iNKT cells, analogous to Treg cells, perform different function at different stages of intestinal cancer.

Quite surprisingly, the characteristics of iNKT cells in polyps of *Apc*^min/+^ mice differed significantly from iNKT cells in lymphoid organs (17). Most importantly, they were negative for PLZF, which has been considered a master transcription factor for NKT cells (39). Polyp iNKT cells also had lower expression of CD4, NK1.1, and CD44, and were enriched for IL-10- and IL-17-producing cells. They also had a lower production of pro-inflammatory cytokines compared with iNKT cells in other organs. These distinct characteristics of intestinal polyp iNKT cells likely underpin their unusual function to suppress tumor immunity.

## The Role of iNKT Cells in the Gut During Intestinal Inflammation and Inflammation-Driven Intestinal Cancer

In the steady state, there does not seem to be a role for iNKT cells to maintain immune homeostasis in the intestine, as to our knowledge, there are no reports of evident inflammation or alteration in the inflammatory microenvironment in the gut in normally housed and unchallenged *Jα18*- or *CD1d*-deficient mice (also, our unpublished data). In contrast, iNKT cells have been shown to be involved in the regulation of inflammation in murine models of induced IBD and induced inflammation-associated colon cancer.

For example, the murine oxazalone-induced colitis model shares immunological features with human ulcerative colitis. It was demonstrated that iNKT cells had an important role in driving inflammation in this model (29) and IL-13 produced by iNKT cells was essential for disease to develop. A similar function was proposed for CD1d-restricted dNKT cells in patients with ulcerative colitis (40). To establish a model for inflammation-induced colon cancer, a chronic form of oxazalone-induced colitis was combined with the genotoxic agent azoxymethane (AOM) resulting in colon tumors. Tumor development was associated with the appearance of F4/80^+^CD11b^high^Gr1^low^ M2 macrophages that produced the tumor-promoting factors IL-6 and EGF, in a CD1d- and IL-13-dependent manner (41). Interestingly, while oxazalone + AOM treatment resulted in robust colitis in mice lacking the major signaling pathway for toll-like receptors (TLR) (MyD88^−/−^ mice), these mice did not develop colon tumors. A dependence on MyD88 for polyp formation has also been demonstrated in *Apc*^min/+^ mice (42), indicating that signaling from the microbial flora plays a major role in tumor development. The immunoregulatory functions of iNKT cells in the oxazalone colitis and the oxazalone + AOM models appear similar to what has been described for iNKT cells in the murine intestinal polyposis model (*Apc*^min/+^ mice) (17). The similarity in regulatory function performed by iNKT cells in these models indicates that tissue location may influence local iNKT-cell behavior.

Both murine and human intestinal epithelial cells express CD1d (43, 44), which upon cross-linking by iNKT TCR mediates retrograde signaling in epithelial cells inducing their IL-10 production (45). Epithelial cell-specific deletion of IL-10 or CD1d leads to more severe oxazalone colitis, illustrating protective anti-inflammatory cross talk between intestinal epithelium and iNKT cells (46). In contrast, IL-13-mediated inflammation in this model was dependent on CD1d expression on bone-marrow-derived cells. Thus, in the oxazalone-induced IBD model, iNKT cells are activated to produce pro-inflammatory IL-13 by CD1d expressed by bone-marrow-derived cells, while interaction with CD1d on epithelial cells results in epithelial cell production of protective IL-10.

A somewhat different picture is found in the dextran sodium sulfate (DSS) colitis model of IBD, which results from disruption of the colonic epithelial barrier. Inflammation in this model differs from that of the oxazalone model in that it is mediated by innate cells and is not dependent on adaptive immunity (47). If present, however, T cells can promote the inflammatory response as exemplified by a TH1 or mixed TH1/2 response has been found in an acute or chronic DSS colitis, respectively. In the acute DSS model, activation of iNKT cells ameliorated disease after administration of αGalCer or OCH, a structural variant of αGalCer that skews the cytokine response toward TH2 (48, 49). It was therefore speculated that the protection was due to anti-inflammatory TH2-type cytokines induced by the activation of iNKT cells. The natural effect of iNKT cells on DSS colitis (without experimental activation of iNKT cells) was not directly addressed in these studies. In another model, repeated administration of DSS has been applied together with AOM to induce inflammation-driven colon cancer development. In these studies, an increased number of colon tumors and more severe inflammation were found in mice lacking iNKT cells compared with control mice (50). Administration of αGalCer, but not the TH2-skewing iNKT-cell ligand OCH, led to reduced inflammation, reduced colon tumor formation and decreased IL-13. This suggests that iNKT cells protected the mice from colitis, and consequently from the downstream development of colon tumors in the AOM-DSS model, through a mechanism that includes suppression of IL-13 production. It is possible that the disruption of the epithelial barrier and leakage of bacteria/bacterial fragments into the intestinal tissue in this model results in an inflammatory microenvironment that is different from that of the oxazalone colitis model. This may influence the function of iNKT cells, as NKT cells can be activated by inflammatory cytokines produced by TLR-stimulated dendritic cells, and IL-12 strongly skews NKT cells toward the production of TH1 cytokines (51).

## Interplay Between iNKT Cells and the Intestinal Microbiota

The commensal intestinal microbiota is essential for the development and function of the host immune system (52). The interplay between the intestinal microbiota and iNKT cells in health and disease was recently discussed in a comprehensive review (53). iNKT cells are established in the intestinal mucosa in an IL-12- and MyD88-independent manner. However, microbial colonization affects the phenotype and function of systemic iNKT cells and increases their immune responsiveness. This occurs independently of MyD88 and is thought instead to depend on agonistic iNKT cell ligands produced by bacteria such as *Sphingomonas* (54). In another study, neonatal microbial colonization limited iNKT-cell numbers in the adult mouse colon, which reduced sensitivity to oxazalone-induced intestinal inflammation (55). This was shown to depend on an abundant inhibitory glycosphingolipid from *Bacteroides fragilis* that bound CD1d but failed to activate iNKT cells (56). At the same time, NKT cells influence the growth of the commensal microflora (57). Mice lacking NKT cells demonstrate an accelerated microbial colonization and an altered composition of the intestinal microbiota. NKT cells also provide protection to bacterial infections, as recently reviewed (51, 58). Thus, iNKT cells are strongly affected by different species of bacteria that colonize the intestine. It is unclear, however, to what extent the intestinal microbial flora can skew the functional program in local iNKT cells, as has been described for conventional T cells (52).

## Suppression of Tumor Immunity by Invariant and dNKT Cells in Other Tumor Models

Similar to their immunosuppression in intestinal polyposis, iNKT cells have been shown to suppress immunity in some other tumor models. However, the mechanisms underlying NKT-cell suppression of tumor immunity has been most exhaustively studied for dNKT cells. A series of elegant publications by Terabe and Berzofsky and coworkers detail how dNKT cells suppressed CD8 T-cell tumor immunity to different transplanted tumors (27, 59, 60). In these models, it was shown that dNKT cells produced IL-13 that activated CD11b^+^Gr-1^+^ myeloid cells to produce TGF-β. This suppressed cytotoxic T-cell activity, resulting in tumor recurrence. Tumor recurrence was prevented in mice deficient of all NKT cells (but not in mice lacking iNKT cells only), or by blocking TGF-β or depleting Gr-1^+^ cells. A similar mechanism may underlie the dNKT-cell suppression of immunity to a B lymphoma where increased levels of IL-13, TGF-β, and myeloid-derived suppressor cells correlated with enhanced tumor growth (28). In contrast, lack of dNKT cells and reduced tumor growth was associated with increased IFN-γ and IL-12. In these models, iNKT cells had a protective effect, suggesting that dNKT cells and iNKT cells counteracted each other in the regulation of immunity to this tumor. In myeloma patients, it has been proposed that also human dNKT cells can have suppressive role in tumor immunity (61).

Interestingly, as suggested from two lymphoma models, sometimes iNKT cells seem to be able to support suppression of tumor immunity by mechanisms similar to those described above for dNKT cells. In a transplantable B-cell lymphoma model it was found that iNKT cells suppressed antitumor CD8^+^ T cells required for lymphoma eradication (19). While the majority of WT mice succumbed to the lymphoma, mice lacking iNKT cells cleared the tumor cells. In another study, the survival of WT mice inoculated with CD1d-transfected T lymphoma RMA-S cells was significantly lower than inoculated *CD1d*^−/−^ and *Jα18*^−/−^ mice (18). Improved survival in iNKT-cell-deficient mice was associated with increased production of IFN-γ, while tumor growth in WT mice correlated with higher IL-13 production. Lymphoma growth in WT mice was not observed after inoculation of untransfected RMA-S cells, suggesting that lymphoma overexpression of CD1d-induced immunosuppressive activities of iNKT cells. Interestingly, studies of patients with B-cell-derived chronic lymphatic leukemia indicate a similar scenario. Here, CD1d expression on tumor cells was higher in patients with unfavorable prognosis, and the level of CD1d on leukemia cells was inversely correlated with iNKT-cell frequencies (26, 62). Higher levels of CD1d on lymphoma cells were also correlated with reduced IFN-γ production by remaining iNKT cells. However, it was not reported in these studies whether these iNKT cells instead produce other cytokines such as IL-10 or IL-13 (26). Thus, in some tumor models, iNKT cells express immunosuppressive effector functions similar to those described for dNKT cells. These studies indicate that suppressive iNKT cells may be more likely to be induced in a situation where the tumor cells express high levels of CD1d.

## Immunosuppression by Adipose Tissue iNKT Cells

The mechanisms that underpin the iNKT-cell-mediated regulation of tumor immunity in the *Apc*^min/+^ model also bear striking resemblance to what has been described in adipose tissue homeostasis (16). iNKT cells are highly enriched in this tissue in both mice and humans, and are suggested to protect against obesity-induced inflammation. It was suggested that in the lean state, production of IL-2 by iNKT cells promoted Treg cells in adipose tissue. Furthermore, mice deficient in iNKT cells had a reduced frequency of Treg cells that proliferated less at this site. It is notable that in the prevention of autoreactivity, iNKT cells have also been shown to support Treg cells in several different settings of autoimmunity and tolerance induction, through a variety of mechanisms (13). Moreover, adipose tissue iNKT cells had high expression of E4BP4, a transcription factor that determines production of IL-10 in T cells (16). iNKT cells were localized juxtaposed to macrophages in adipose tissue, and IL-10 produced by activated iNKT cells enhanced M2 and reduced M1 phenotype in adipose tissue macrophages. This strongly suggests that comparable subsets of regulatory iNKT cells regulate immunity by very similar mechanisms in intestinal polyps of *Apc*^min/+^ mice and in adipose tissue of lean WT mice.

## The PLZF-Negative/Adipose/iNKT10 Subset of iNKT Cells with Immunosuppressive Capacities

A unique feature in adipose tissue iNKT cells (16) that is shared with intestinal polyp iNKT cells (17) is a lack of the NKT-cell transcription factor PLZF. In fact, adipose iNKT cells demonstrated some similarities to the reduced population of iNKT cells that remain in PLZF-deficient mice (16). Moreover, polyp and adipose iNKT cells share the production of IL-10. IL-10-producing iNKT cells, then coined iNKT10 cells, were also increased in αGalCer-injected mice, and were enriched in adipose tissue after injection of αGalCer (15). This latter study showed that a single treatment of mice with αGalCer led to expansion of IL-10-producing iNKT cells concomitant with reduced production of pro-inflammatory cytokines by iNKT cells. Strikingly, the αGalCer-induced iNKT10 cells could suppress tumor immunity and ameliorate experimental autoimmune encephalomyelitis (15). These iNKT10 cells thus appear similar to adipose iNKT cells and intestinal polyp iNKT cells. Taken together, these cell types seem to constitute a unique regulatory subset(s) of iNKT cells that is not yet well defined, and that has not been taken into account in previous studies.

## Differentiation of Suppressive iNKT Cells—Thymically Determined or an Effect of the Local Microenvironment?

It is yet to be revealed what determines the development of immunosuppressive PLZF-negative iNKT cells, and how these cells localize to the tissue where they suppress immunity. Do these cells develop during thymic maturation followed by selective homing to specific tissues? Or is their function influenced during peripheral activation by tissue-derived signals such as those from local CD1d-expressing cells and other cues in the tissue microenvironment? A recent study found that mutating a hydrophobic patch in the iNKT-cell TCR resulted in thymic selection of functionally altered iNKT cells that accumulated in adipose tissue (63). In the thymus, these iNKT cells already expressed some characteristics of adipose iNKT cells. This suggests that iNKT cells with the immunosuppressive PLZF-negative phenotype may be committed during thymic development, as has been proposed for other functional subsets of iNKT cells (14). If this is the case, there may be a selective expression of homing receptors on this subset of iNKT cells that guide their localization to adipose tissue and intestinal polyps or other tumor or inflammatory sites where they may suppress immunity. Adipose tissue-specific recruitment is unlikely to occur constitutively at a high rate, however, as adipose iNKT cells are mostly resident and non-recirculating (16). On the other hand, it is also likely that the tissue microenvironment will alter the function of immune cells that enter the tissue (64). Further transcriptomic and epigenetic analysis of iNKT-cell subsets will provide important information in this respect (65). The finding that the immune response to syngeneic colon cancer cells is significantly different when the cells grow in the intestine compared with subcutaneous growth demonstrates that the immune microenvironment varies between tissues (66). In the *Apc*^min/+^ model, we showed that transfer of hepatic iNKT cells predominantly of the iNKT1 type to iNKT-cell-deficient *Jα18*^−/−^*Apc*^min/+^ mice reconstituted immunosuppressive effects of iNKT cells in intestinal polyps (17). This indicates that the polyp microenvironment might alter the function of iNKT cells. Highly relevant in this context is the recent interest in immunometabolism and investigations of how the metabolism of immune cells is modulated in fat tissue and tumors, resulting in alterations of immune cell function (67, 68). It is important to investigate whether metabolic states of iNKT cells correlate with their programs and their immunosuppressive activities in different situations.

## Activation of Immunosuppressive iNKT Cells—A Role for the CD1d-Expressing Cell and CD1d-Presented Ligands?

It seems feasible that a major importance for determining iNKT-cell function will be attributed to the CD1d-expressing cell type that activates iNKT cells in the periphery. Induced effector functions are likely different if CD1d is presented to iNKT cells on a professional antigen-presenting cell such as dendritic cells, which may produce pro-inflammatory cytokines like IL-12 and provide other accessory signals, or if iNKT cells are activated by CD1 expressed by non-professional antigen-presenting cells such as adipocytes, epithelium, or tumor cells of different origin. Activation by non-professional antigen-presenting cells may be more likely to induce an anti-inflammatory function in iNKT cells. In the oxazalone-induced colitis model, iNKT cells were induced to produce IL-13 by bone-marrow-derived cells. In contrast, IL-13 production was reduced by iNKT-cell interaction with CD1d-expressing intestinal epithelial cells through induction of IL-10 secretion by the epithelial cells (46). The adipose tissue iNKT cells expressed high levels of Nur77 (16), a nuclear receptor that is upregulated upon TCR ligation. This suggests that adipose iNKT cells may be under constant TCR stimulation. Adipocytes express high levels of CD1d that is required for iNKT-cell regulation of immune homeostasis in adipose tissue (64), indicating that adipocytes act as non-professional antigen-presenting cells for iNKT cells. Whether adipose tissue-specific signals or CD1d-presented lipids determine the functional phenotype of adipose iNKT cells remains to be determined. Interestingly, a role for CD1d on tumor cells was also suggested (18, 26, 62). CD1d expression on tumor cells was associated with the induction of immunosuppressive functions in iNKT cells or the induction of “non-functional” iNKT cells.

The activating ligand presented on CD1d may also play a role in selective induction of iNKT subsets and functions. Considering the dynamic nature of glycolipid metabolism and modulations of these processes in activated or stressed cells, it seems feasible that cancer cells contain an altered set of lipids that are potential iNKT ligands. Such ligands could be presented on CD1d, either on the cancer cells themselves, if CD1d-positive, or on antigen-presenting cells and result in different outcomes. An intriguing example is the disialoganglioside GD3. This is expressed only in a few normal tissues at low levels but accumulates in human melanoma, and in ascites fluid in ovarian cancer patients (69, 70). Using a mouse model, one study found that GD3 was an activating ligand for a small subset of iNKT cells that was only detectable in immunized mice (69). Interestingly, these iNKT cells produced IL-4, but not IFN-γ, in response to immunization with GD3-pulsed dendritic cells. Another study found that GD3 bound both human and mouse CD1d with high affinity (70). However, in contrast to the previous study, GD3 was not found to stimulate iNKT cells. It rather inhibited αGC stimulation of iNKT cells *in vitro* and *in vivo*, and GD3-loaded CD1d multimers did not bind iNKT cells. The latter study may have missed the small GD3-reactive iNKT-cell subset, as these cells were not detectable in non-immunized mice. Thus, GD3 enriched in some cancers seems to prevent induction of TH1 tumor immunity by iNKT cells in two ways: it inhibits the majority iNKT cells from activation with agonist ligands by binding to CD1d with high affinity, while at the same time stimulating a small subset of GD3-specific iNKT cells to secrete IL-4. Another glycolipid that inhibited iNKT-cell activation, gangliotriaosylceramide, was found to be shed from a T-cell lymphoma line (71). Besides these examples, there is little information available about the contribution of CD1d-presented lipids that are cancer specific or upregulated in cancer to activation of NKT cells in tumor immunity.

Research ongoing in many laboratories explore and refine iNKT-cell-directed immunotherapy strategies using αGalCer and structurally related ligands that have an improved induction of tumor immunity, often by the enhancement of TH1 cytokines [for recent reviews, see Ref. (72, 73)]. However, many factors influence the outcome of iNKT-cell activation in tumor immunity. For example, Wingender et al. demonstrated that αGalCer as well as the TH1-biasing structural variant of αGalCer, C-glycoside, could induce the expansion of iNKT10 cells, while a TH2-biasing variant, OCH, did not induce their expansion (74). Another study identified β-mannosylceramide as a ligand for mouse and human iNKT cells (75). Administration of β-mannosylceramide protected mice from tumors in a manner that was dependent on TNF-α and nitric oxide synthase and only partially dependent on IFN-γ. In contrast, protection afforded by αGalCer was completely dependent on IFN-γ. To investigate whether iNKT-cell-targeted immunotherapy with agonist ligands could counteract the immunosuppressive effects of iNKT cells in the *Apc*^min/+^ intestinal polyposis model, we treated mice repeatedly with αGalCer or the TH2-biasing structural variant of αGalCer, C20:2 (17). Despite the natural tumor-promoting effect of iNKT cells in untreated mice, treatment with αGalCer resulted in decreased polyp numbers. This suggests that αGalCer may either modulate the function of polyp iNKT cells, or overcome their immunosuppressive effect by activating iNKT cells systemically. As expected, mice treated with C20:2 had increased numbers of polyps. This is consistent with a TH2 cytokine profile induced by this ligand, resulting in suppression of a TH1 antitumor immune response. Quite surprisingly, treatment with the TH1-biasing ligand C-glycoside had no effect on polyp counts (76). As C-glycoside has been shown to induce TH1 cytokines, this treatment would be expected to reduce polyp counts, and be even more effective than αGalCer. It is possible that the lack of effect on polyps after repeated treatment of *Apc*^min/+^ with C-glycoside is a consequence of expansion of iNKT10 cells by this ligand, as demonstrated by Wingender and coworkers (74). Our own data from the *Apc*^min/+^ model also show that different time periods of treatment with iNKT ligands can result in opposite effects (76). Taken together, these data show that modulation of tumor immunity by treatment with iNKT-cell ligands is not always predictable. They also warrant some caution and motivate further studies of the effects of iNKT-cell-directed therapy on iNKT-cell function and downstream regulation of tumor immunity.

## Concluding Remarks and Outstanding Questions

Natural killer T cells are important players in the regulation of tumor immunity. iNKT cells are an expanded subset of NKT cells with potent and rapid effector response that show great promise as targets for tumor immunotherapy. However, the effect of iNKT cells, activated naturally or by artificial ligands, on tumor immunity is influenced by many factors. This makes the outcome of iNKT-cell-targeted therapy difficult to predict. The recent identification of a distinct subset of iNKT cells with immunosuppressive properties, shown to suppress tumor immunity and promote tumor growth, calls for more detailed investigation of iNKT cells in different cancer settings. There are a number of outstanding questions that will be important to resolve to clarify the differentiation, activation, and function of immunosuppressive iNKT cells. Is the lack of PLZF a common feature of immunosuppressive iNKT cells in different tumor settings? Is this also the case for regulatory iNKT cells in inflammation and autoimmune diseases? Is the PLZF-negative functional phenotype determined during thymic development, or is imposed by activation and/or the tissue microenvironment under specific conditions? What is the role of the tumor microenvironment, local cytokines, and other signals in modulating the iNKT-cell phenotype, and in the attraction of functionally different iNKT-cell subsets? What is the role of the CD1d-expressing antigen-presenting cell for induced immunosuppressive iNKT-cell functions? What is the effect of iNKT-targeting immunotherapy on tumor-associated iNKT cells? What is the influence of the intestinal microbiota and inflammation on local iNKT cells? Undoubtedly, our knowledge regarding these issues will be significantly expanded in the coming years, and will hopefully contribute to improved strategies for iNKT-cell-directed tumor immunotherapy.

## Author Contributions

Both authors contributed substantially to the work and approved it for publication.

## Conflict of Interest Statement

The authors declare that the research was conducted in the absence of any commercial or financial relationships that could be construed as a potential conflict of interest.

## Abbreviations

AOM, azoxymethane; DSS, dextran sodium sulfate; IBD, inflammatory bowel disease; NKT, natural killer T; dNKT, NKT cell with diverse TCR; iNKT, NKT cell with invariant TCR; TCR, T-cell receptor; TH, T helper; TLR, toll-like receptor; Treg, regulatory T.
